# Seasonal and developmental stage changes in mucilage carbohydrate content shape the kelp microbiome

**DOI:** 10.1093/ismeco/ycaf197

**Published:** 2025-10-29

**Authors:** Chance J English, Meenakshi Manoj, Lillian C Henderson, Keri Opalk, Craig A Carlson

**Affiliations:** Marine Science Institute, University of California, Santa Barbara, Santa Barbara, CA 93106, United States; Earth Research Institute, University of California, Santa Barbara, Santa Barbara, CA 93106, United States; Marine Science Institute, University of California, Santa Barbara, Santa Barbara, CA 93106, United States; Marine Science Institute, University of California, Santa Barbara, Santa Barbara, CA 93106, United States; Marine Science Institute, University of California, Santa Barbara, Santa Barbara, CA 93106, United States; Marine Science Institute, University of California, Santa Barbara, Santa Barbara, CA 93106, United States; Department of Ecology, Evolution and Marine Biology, University of California, Santa Barbara, Santa Barbara, CA 93106, United States

**Keywords:** microbiome, macroalgae, kelp, dissolved organic carbon, carbohydrates, Planctomycetota, alginate, fucoidan, mucus

## Abstract

A large amount of a photoautotroph’s fixed carbon is released as dissolved organic matter, from both exudation and solubilized detritus. This dissolved material contributes to a surface mucilage layer that shapes their immediate environment, including the composition of their microbiome. Here we evaluated the microbiome and mucilage carbohydrate composition of *Macrocystis pyrifera* (giant kelp), a globally distributed foundation species, in response to seasonal nutrient availability and developmental stage. We combine 16S rRNA amplicon analysis of the giant kelp microbiome with carbohydrate monomer analysis of kelp mucilage to examine microbe-mucilage relationships. We found significant differences in the microbiome and mucilage composition between seasons and developmental stages of giant kelp. Higher tissue-nitrogen content in the spring coincided with elevated amounts of glucosamine, a nitrogen-containing sugar, in giant kelp mucilage, while senescence led to the release of mannuronic acid, an alginate indicator. The release of glucosamine and fucose-rich mucilage was correlated with an increase in the relative abundance of bacteria within the Planctomycetota phylum, whereas mannuronic acid-rich mucilage coincided with an increase in the relative abundance of members of the Flavobacteriia and Gammaproteobacteria lineages. We investigated putative carbohydrate-microbe relationships by isolating a member of the Planctomycetota phylum from the surface of giant kelp. Using whole genome analysis and growth assays, we demonstrate that this isolate grows on fucoidan and *N-*acetyl glucosamine, but not alginate, consistent with the observed relative abundance of this clade in the kelp microbiome in response to variable mucilage carbohydrate content. This suggests a key role of kelp mucilage carbohydrate composition in structuring its microbiome as has been observed for other organisms such as corals and within the human gut.

## Introduction

Primary producers, such as plants and macroalgae, have evolved in environments surrounded by microorganisms and host-associated microbes can improve the fitness and growth of the host through defense, nutrient acquisition, and hormone production [1–5]. A growing body of evidence indicates that autotrophic organisms regulate their microbiome by exuding metabolites that promote microbial growth or inhibit the growth of pathogens [6, 7]. The exudate and host-microbe associations are dynamic and can vary in response to the host’s developmental stage and abiotic stressors [8, 9]. Unraveling the relationship between host physiology, exudate composition, and the microbiome structure can provide a mechanistic understanding of the establishment and function of the microbiome of primary producers.

Carbohydrates, including simple sugars, disaccharides, and complex polysaccharides, are typically the dominant organic material exuded by primary producers [10, 11]. The active exudation of carbon-rich organic matter by organisms was originally proposed as an “overflow” mechanism that allowed photosynthesis to continue when light and nutrients needed for biosynthesis became uncoupled [12, 13]. More recently, it has been proposed that the exudation of water-soluble carbohydrate-rich material serves to form a surface mucilage layer that prevents the attachment of pathogens [7], promotes the growth of beneficial microbial partners [9, 14] or aids in the transport of energy and nutrients to the organism [15]. The potential role of carbohydrate exudation in shaping microbiomes is evidenced in studies of corals that reveal significant correlations between the host’s mucilage content and the relative abundance of certain microbial taxa [16, 17]. The composition of mucilage carbohydrates by corals is species-specific [18, 19] and varies in response to abiotic factors such as temperature [16]. While these relationships are established for corals, the role of carbohydrate exudation in shaping the microbiome has yet to be evaluated for important foundation species such as kelps.

Kelp forests are critical marine habitats that occupy a third of the world’s coastline [20]. In addition to their contributions to biodiversity [21] and global economies [22], it is hypothesized that kelps contribute to carbon sequestration through the export of dissolved organic carbon (DOC) and detrital particulate carbon [23–25]. In this context, the decline of kelp forests [26] due to stressors such as ocean warming is a serious threat to ecosystem function, marine carbon cycling, and economies. This has prompted calls for large-scale intervention through natural kelp forest restoration [27] or aquaculture to offset these losses and increase current production yields [28]. Therefore, identifying and understanding the underlying factors for kelp health and resilience is of critical importance for monitoring, management, and restoration of these ecosystems, their services, and the economies dependent on kelp.

Decades of research have focused on understanding the impact of environmental factors, such as light, nutrients, wave disturbance, and temperature on kelp growth [29–33]. In temperate coastal areas, such as California, kelp growth rates and physiology are influenced by seasonal nitrate availability. Low seawater nitrate concentrations (NO_3_^−^ < 1 μM) throughout the summer and fall result in elevated tissue carbon to nitrogen ratios and when tissue nitrogen content falls below 1% of dry mass, kelp survival can become compromised [33]. In comparison to abiotic factors, less is known about how the kelp microbiome affects kelp growth and resilience, although there is a growing understanding of the potential role of microbes in kelp health and resistance to disease and herbivory [34–36]. Several studies demonstrate that kelp harbor a microbiome that is distinct from the surrounding seawater [37–39] and genomic analysis of kelp microbiomes suggests potential positive interactions, such as antibacterial defense, or vitamin acquisition [40, 41], between kelp and specific microbial lineages such as Planctomycetota. Further, there are significant associations between environmental conditions (i.e. temperature, nutrients), the physiological condition of kelp, its productivity, and its microbiome composition [42–44]. However, the underlying mechanisms between kelp physiology and microbiome composition are unresolved, although previous studies suggest exudate composition, environmental perturbations, or the interaction between the two may be important [3, 45, 46].

In this study, we aimed to understand how the composition of kelp mucilage shapes the microbiome of *Macrocystis pyrifera*, hereafter giant kelp. We hypothesized that abiotic factors (NO_3_^−^ availability) and developmental stage would alter the chemical composition of kelp mucilage and that these altered chemical profiles would shape the giant kelp microbiome. We sampled tagged cohorts of giant kelp fronds to track the development of their microbiome across developmental stages and between the warm, nitrate-depleted summer, and the spring upwelling period when nitrate is replete. Simultaneous measurements of mucilage carbohydrate composition and 16S rRNA gene analysis were employed to identify microbe-mucilage associations. We observed significant correlations between abundant microbial taxa and the carbohydrate content of giant kelp mucilage. To test some of these associations, we isolated a member of the Planctomycetota phylum, a dominant kelp-associated bacterial group. Using whole genome sequencing, phylogenomic analysis and growth assays, we demonstrate that the carbohydrate substrate preferences of this clade reflect its relative abundance patterns in the kelp microbiome. This work suggests an important role for carbohydrates in shaping the kelp microbiome.

## Materials and methods

### Sampling, kelp blade age and developmental stage, and environmental variables

Giant kelp blades were sampled from two tagged cohorts at Mohawk Reef (34° 23.6600′ N, 119° 43.8000′ W) in the Santa Barbara Channel, CA, USA in the summer of 2023 and spring of 2024. Cohorts were tagged by placing a cable tie around the stipe 2 m back from the apical meristem of 100–200 growing fronds. At intervals of 2–3 weeks, single blades (6–32 g wet weight) from six random fronds were sampled to measure their tissue nitrogen (tissue-N), exudate carbohydrate composition, and microbiome. The developmental stage of kelp blades was categorized as either “mature” or “senescent” if they were younger than or older than 50 days old, respectively [30]. Blades were collected in the morning (08:00–10:00) and transported in a plastic cooler filled with ambient seawater to a near-shore laboratory, where they were incubated in 10 l acrylic tanks filled with filtered (0.2 μm) seawater. Incubations were started between 10:00–12:00 and maintained at *in situ* temperature (Summer: 17–19°C, Spring: 12–14°C) using a temperature-controlled water bath for 6–9 h. Incubation tanks were equipped with magnetic stir bars to maintain water circulation. Light levels were adjusted every 2–3 h, decreasing from saturating intensity (1140–1517 μmol photons m^−2^ s^−1^) to darkness (0 μmol photons m^−2^ s^−1^) to simulate the transition from day to night. Duplicate DOC (*n* = 84) and one hydrolyzable carbohydrate (*n* = 42) sample were collected by siphon at the beginning and end of each incubation and filtered through pre-combusted glass-fiber filters (Whatman GF-75, 0.3 μm). Following their incubation, kelp blade tissue-N was sampled as a measure of nitrogen stress and developmental stage. For tissue-N analysis, giant kelp blades were scraped cleaned of epibionts with a metal spatula, dried at 60°C for 3 days and then ground to a fine powder before analysis of a 10 mg subsample with an Exeter Analytical CE-440 CHN/O/S elemental analyzer.

Environmental conditions at Mohawk Reef during our study were evaluated using bottom temperature data collected by the Santa Barbara Channel Long-Term Ecological Research program [47]. Average daily temperature was calculated from submersible temperature loggers at Mohawk Reef. Nitrate concentrations were calculated from temperature using an exponential “T2N” relationship derived from an inshore temperature and nitrate dataset [48] (Supplementary Fig. S1).

### DOC production and mucilage carbohydrate composition

DOC analysis was carried out following established protocols [49]. Briefly, duplicate DOC samples were filtered into pre-combusted 40 ml Environmental Protection Agency (EPA) borosilicate vials with polytetrafluoroethylene (PTFE) lined caps, acidified to pH < 3 with the addition of 60 μl of 4 N HCl and analyzed using a Shimadzu Total Organic Carbon (TOC)-V or TOC-L.

As mucilage is water-soluble and continuously shed, we measured the accumulation of dissolved carbohydrates from incubation seawater samples taken alongside DOC samples. Samples were stored frozen at −20°C until analysis by high-performance anion exchange chromatography with pulsed amperometric detection (HPAEC-PAD). We followed established dialysis and HPAEC-PAD gradients [50]. Briefly, samples were dialyzed using Spectra/Por 7 tubing (1000 Da) against ultrapure water and then hydrolyzed in duplicate for 20 h at 100°C in 0.4 M HCl then neutralized under a flow of N_2_ gas. Samples were run on a DIONEX ICS5000+ and sugars were separated using a Carbopac PA10 column (4 × 250 mm) with a Carbopac PA10 guard column (4 × 50 mm). Neutral and amino sugars were eluted with 18 mM NaOH followed by 100 mM NaOH/200 mM Na-Acetate to elute acidic sugars. The system was calibrated using a common standard sugar mix [18, 51] dissolved in ultrapure water containing fucose (1 mM), rhamnose (1 mM), arabinose (1 mM), galactosamine (0.25 mM), glucosamine (0.25 mM), galactose (1 mM), glucose (1 mM), mannose + xylose (2 mM), galacturonic acid (1 mM), glucuronic acid (1 mM), and mannuronic acid (0.5 mM). Linearity of the calibration curves were observed for concentrations ranging from 10 nM to 1 μM. In our dissolved carbohydrate samples, we observed glucose and mannose + xylose contamination due to leaching from the Spectra/Por 7 dialysis tubing, so these sugars were removed from subsequent analysis. Previous studies demonstrate these sugars are not substantial components of carbohydrates released by brown macroalgae [18, 52].

Principal component analysis was used to visualize changes in the composition of hydrolyzable sugar monomers in giant kelp mucilage. Sugars were normalized to molar percentages (mole%) relative to other sugars. Statistical analysis of the differences in the composition of the carbohydrates released by giant kelp between the two seasons and developmental stages was evaluated by permutational multivariate analysis of variance (PERMANOVA) using the *adonis* function in R (vegan 2.7-1) [53] on the scaled mole% of individual sugar monomers.

### Microbiome DNA extraction, 16S rRNA gene sequencing, and amplicon analysis

Following their incubation, but before their preparation for tissue-nitrogen analysis, kelp blades were rinsed with 0.1 μm filtered sterilized seawater and sampled across the entire surface by scraping with sterile cotton-tip swabs (Puritan®) for 30 s. Swabs were placed in sterile 2 ml cryogenic tubes and frozen at −80°C. DNA samples were lysed in sucrose lysis buffer (750 mM Sucrose, 20 mM ethylenediaminetetraacetic acid (EDTA), 400 mM NaCl, 50 mM Tris–HCl, pH = 8.0) with 1% sodium dodecyl sulfate (SDS) and 0.2 mg ml^−1^ proteinase-K at 37°C for 30 min for cell lysis and protein digestion, then at 55°C for 30 min to complete the lysis and degradation of cellular components. DNA was purified using the phenol-chloroform method [54]. For amplification, we targeted the V4 region of the 16S rRNA gene using the 515F-Y (5′-GTGYCAGCMGCCGCGGTAA-3′) and 806RB (5′- GGACTACNVGGGTWTCTAAT-3′) primers (84% coverage *in silico* of 16S rRNA sequences; SILVA TestPrime 1.0) with custom adaptors [55]. Polymerase chain reaction (PCR) was carried out in 25 μl reactions with 12.5 μl KAPA Robust Hotstart ReadyMix (final concentrations: 0.2 mM each dNTP, 1 U DNA Polymerase, 2 mM MgCl_2_), 1μl each of the forward and reverse primers (final concentrations: 0.4 μM), 2 μl of bovine serum albumin (final concentration: 1.6 μg μl^−1^), 6.5 μl of PCR-grade water, and 2 μl of sample DNA and cycled for 3 min at 95°C, followed by 30 cycles of 30 s at 95°C, 30 s at 57°C and 1 min at 72°C, ending with 10 min at 72°C. Four negative controls (PCR-grade water) and two mock community samples were included in the PCR run for quality control. Following PCR, amplified DNA was quantified (Qubit, Thermo Fisher) and manually normalized by pooling equal nmol amounts of DNA from each PCR reaction well. Pooled DNA was concentrated using Amicon® Ultra 0.5 ml 30 K centrifugal filters (Millipore) then non-target DNA bands were removed by gel electrophoresis and extraction. Samples were sequenced on an Illumina MiSeq PE250 and demultiplexed, with zero barcode/index mismatches allowed, at the University of California, Davis DNA Technologies Core.

Demultiplexed raw sequence reads were quality checked and processed using DADA2 [56] in R. Forward and reverse reads were trimmed to 240 and 150 bp, respectively. Trimmed and error corrected reads were dereplicated and pair-end reads were merged and assigned taxonomy using the SILVA database (v183.1 w/species) [57]. In all samples, amplicons identified as mitochondria (0–100 reads, mean = 18 reads) or chloroplasts (143–20 725 reads, mean = 3216 reads) were removed. PCR-water and mock community controls were checked to confirm consistent amplification and lack of contamination and then removed from further analysis (Supplementary Data SS1). Quality filtering of chimeric sequences or sequences with ambiguous bases resulted in 4.4 × 10^6^ sequences from 54 samples. Samples had a read depth from 25 077 to 106 379 sequences, averaging 81 976 and were not rarefied for further analysis [58]. We analyzed differences in kelp microbiome composition by season (summer vs. spring) and developmental stage (mature vs. senescent).

For multivariate analysis, singleton and doubleton amplicon reads were removed and remaining amplicon sequence variants (ASVs) were converted to relative abundances and normalized using a variance stabilizing transformation. For beta diversity calculations, samples were assigned non-metric multidimensional scaling (NMDS) scores based on Bray–Curtis dissimilarities using the *metaMDS* function in phyloseq [59] and differences in community composition between seasons and developmental stages were tested using a PERMANOVA with the *adonis* function in the R [53]. For Shannon diversity calculations, performed with phyloseq, singleton and doubleton amplicons were not removed. Significant differences in the mean relative abundances of amplicons by season and developmental stage were evaluated using the “DEseq2” package in R [60]. Differentially abundant ASVs were those that displayed a log2 fold-change >1.5 between developmental stage or season and a significant false discovery rate (FDR)-adjusted *P*-value (≤.01).

To identify relationships between the giant kelp microbiome and the mucilage carbohydrate composition, we used hierarchical clustering of Spearman’s rank correlations between the relative abundance of significantly differentially abundant ASVs and the mole% of sugar monomers simultaneously released by giant kelp blades. This clusters ASVs based on their degree of correlation with the sugar content of kelp mucilage and allowed us to identify positive or negative microbe-carbohydrate relationships. Hierarchical clustering was arranged using Ward’s minimum variance method with the *pheatmaps* [61] package in R.

### Planctomycetota cultivation, genome sequencing and growth on model carbohydrates

We enriched and isolated a strain of Planctomycetota following the suggestions of Lage and Bondoso [62] and Wiegand *et al.* [63]. Agar plates were prepared with 0.1 μm filtered seawater with 1% agar. Seawater was autoclaved with agar then supplemented with 1 ml l^−1^ vitamin solution (Biotin: 2 mg l^−1^, Folic acid: 2 mg l^−1^, Pyridoxamine-HCl: 10 mg l^−1^, Thiamine-HCl × 2H_2_O: 5 mg l^−1^, Riboflavin: 5 mg l^−1^, Nicotinic acid: 5 mg l^−1^, D-ca-pantothenate: 5 mg l^−1^, Cyanocobalamine: 0.1 mg l^−1^, p-Aminobenzoic acid: 5 mg l^−1^, Lipoic acid: 5 mg l^−1^, KH_2_PO_4_: 900 mg l^−1^) and 1 ml l^−1^ trace metal solution (H_3_BO_3_: 2860 mg l^−1^, MnCl_2_ × 4H_2_O: 1810 mg l^−1^, FeCl_3_ × 6H_2_O: 316 mg l^−1^, ZnSO_4_ × 7H_2_O: 222 mg l^−1^, Na_2_MoO_4_ × 2H_2_O: 390 mg l^−1^, CuSO_4_ × 5 H_2_O: 79 mg l^−1^, Co(NO_3_)_2_ × 6H_2_O: 49 mg l^−1^). For a carbon and nitrogen source, we added 10 ml of 5% *N*-acetyl glucosamine. To prevent fungal growth and select for Planctomycetota, the medium was supplemented with 20 mg l^−1^ of econazole nitrate, 200 mg l^−1^ampicillin, and 1 g l^−1^ streptomycin. Bacterial inoculum was scraped from the surface of a giant kelp blade using a sterile swab for 30 s and resuspended in sterile medium without agar. The cell suspension was filtered through a 5.0 μm filter and enumerated using a GUAVA easycyte HT flow cytometer (Millipore). To each agar plate, we added ~2000 cells. Cell suspensions were streaked across the agar plates and incubated at 20°C until colonies were formed. After 1 month, colonies showing characteristics of Planctomycetota (pink pigmentation, budding cell growth) were selected for restreaking. Candidate colonies were restreaked three times to ensure isolation and then identified using 16S rRNA gene metabarcoding of the V4 region using the SILVA Ref NR SSU r138.1 database. Whole-genome sequencing was performed on the Illumina NovaSeq X Plus platform, producing 2 × 151 bp paired-end reads (Seqcenter, Pittsburgh, PA). Trimmomatic (v0.39) and RQCFilter (BBTools v38.22) were used to remove low-quality or short reads. Reads were assembled and annotated with SPAdes (v3.15.3) and Prokka (v1.14.5), respectively, in the KBase platform [64]. Completion (99.93%) and contamination (1.18%) was checked using CheckM v1.0.18. For phylogenomic analysis, 13 publicly available isolate genomes from the *Pirellulaceae* family were selected from NCBI’s GenBank database and compared to our assembled genome (Supplementary Data SS2). The genome of *Planctomicrobium piriforme* from the Planctomycetota order Planctomycetales was selected as the outgroup. A maximum-likelihood tree was constructed with GToTree [65] using 74 bacteria marker genes and visualized using the iTOL server. Carbohydrate-active enzymes associated with fucoidan and alginate degradation were predicted for our isolate and reference isolates using a set of Hidden Markov Models (HMMs) from the dbCAN2 CAZy collection using HMMER 3 software [66, 67]. Secondary metabolite production potential was analyzed using AntiSMASH [68].

We tested the ability of the *Rhodopirellula sp*. isolate to grow on model carbohydrates in batch cultures. We prepared artificial seawater (ASW) as incubation medium (0.42 M NaCl, 0.029 M Na_2_SO_4_, 0.01 M KCl, 1 mM KBr, 0.05 mM H_3_BO_3_, 0.07 mM NaF,0.05 M MgCl_2_, 0.01 M CaCl_2_, 0.06 mM SrCl_2_, 2.5 mM NaHCO_3_). Medium salinity and pH were adjusted to 33 ppt and 7.5, respectively and then autoclaved. Sterile ASW was amended with 0.1 μm filter sterilized vitamin and trace metal solutions (described above, 1 ml l^−1^) and inorganic nutrients (10 mg l^−1^ NH_4_Cl, 1 mg l^−1^ KH_2_PO_4_). Triplicate 50 ml incubations were prepared for the following treatments: no amendment, *N*-acetyl glucosamine (NAGA; 25 mg l^−1^), alginate (25 mg l^−1^), and fucoidan (25 mg l^−1^). Because commercially available fucoidan is typically contaminated, notably by alginate and polyphenols [69, 70], we used ion exchange chromatography (IEX) to purify fucoidan sourced from giant kelp (Sigma-Aldrich, product #F8065). Purification was performed using a medium-scale IEX setup described by Sichert *et al.* [70]. The composition of the purified fucoidan was checked by ^1^H-^13^C heteronuclear single quantum coherence nuclear magnetic resonance (HSQCNMR) and HPAEC-PAD (Supplementary Figs. SS2–SS4). Alginate (Sigma-Aldrich PHR1471) and *N-*acetyl glucosamine (Fisher Scientific ICN100068) were certified to be 88% and 100% pure, respectively. An isolate colony was resuspended in sterile ASW without amended carbohydrates. After gently shaking for 20 min, the cell suspension was 5.0 μm filtered and enumerated using a GUAVA easycyte HT flow cytometer (Millipore). Cells were added to incubation vessels at a concentration of ~6.0 × 10^4^ cells ml^−1^. For all treatments, triplicate control incubations without cells were sampled to confirm sterility of the incubation media and carbohydrate amendments. Incubation cell counts were determined by flow cytometry (GUAVA easycyte HT, Millipore) daily from 0 to 5 days and at 9 days. For flow cytometry sample preparation, 1 ml of culture was fixed with paraformaldehyde (5% final concentration) before a 198 μl subsample was aliquoted into a 96-well plate and stained with 2 μl of a SYBR-Green 1 (1:50 dilution in TE buffer of a 10 000× commercial stock).

## Results

### Kelp physiological response to seasonal nitrate availability and developmental stage

**Figure 1:**
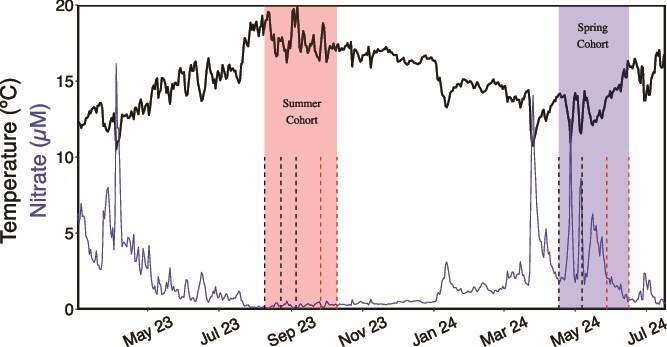
Mohawk Reef environmental conditions and sampling dates in this study. Daily average temperature and nitrate concentrations between January 2023 and August 2024. Shaded red and blue regions show the sampling periods for the summer and spring cohorts used in this study, respectively. Vertical dashed lines extending from the x-axis mark the sampling dates within each cohort. The black and red color of the dashed lines indicate sampling of kelp that was in a mature (<50 days) or senescent (>50 days) development stage, respectively.

**Figure 2:**
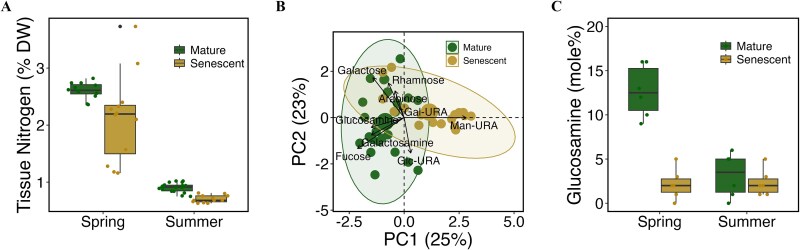
Seasonal and developmental stage changes in giant kelp physiology and mucilage carbohydrate composition. (A) Giant kelp tissue nitrogen content as a percentage of dry weight between mature and senescent phase kelp in spring and summer. (B) Principal component (PC) analysis of giant kelp mucilage carbohydrates as molar percentages between mature and senescent phase kelp. Percentages is parenthesis on each axis represent variances explained by each PC. Ellipses represent 95% confidence regions between mature and senescent kelp. Arrow lengths represent the strength of the correlation between each individual sugar monomer to the two PCs (PC1 and PC2) shown. Large symbols in center of the ellipses are the centroids. Sugar monomer names are overlaid next to arrows. Glc-URA, glucuronic acid); Gal-URA, galacturonic acid; Man-URA, mannuronic acid. (C) Mole% of glucosamine in total moles of sugars produced by giant kelp between developmental stages and seasons. Boxplots: The top and bottom border of each box represent the 25th and 75th percentiles, the horizontal line inside each box represents the median and the whiskers represent the range of the points (1.5 × interquartile range (IQR)) excluding outliers. Outliers are shown by black points outside the whiskers. Colored points show sample values for each boxplot.

We observed variations in kelp developmental stage driven by seasonal nitrate availability and age. In both seasons, rapid changes in physiology occurred after kelp blades passed 50 days of age, which we use to demarcate the transition from maturity to senescence. Across all samples, tissue-N (*g*_Nitrogen_/*g*_Dry weight_ × 100) ranged from 0.63% to 3.7%. Mature kelp sampled in the spring had a significantly higher tissue-N content than mature summer kelp due to higher seawater nitrate concentrations (Fig. 1, Fig. 2A; Welch’s t-test: t = −22.6, df = 19.0, *P* < .001). The average (±1 SD) tissue-N of mature kelp blades in the summer and spring were 0.92% ± 0.08% and 2.6% ± 0.14%, respectively (Fig. 2A). After entering the senescent phase, average (±1 SD) tissue-N decreased to 0.7% ± 0.06% and 2.1% ± 0.77% in the summer and spring, respectively (Fig. 2A).

### Seasonal and developmental stage-driven changes in exudate carbohydrate composition

Hydrolyzable carbohydrates accounted for 10.3% ± 4.9% of giant kelp produced DOC across all incubations, regardless of season or age. We observed a significant difference in the carbohydrate sugar content of kelp mucilage between the mature and senescent developmental stages (Fig. 2B; PERMANOVA, *R*^2^ = 0.14, *P* < .001) and between seasons (PERMANOVA, *R*^2^ = 0.07, *P* = .006). Differences in sugar content between developmental stages were driven by the change in mole% of fucose and mannuronic acid (Fig. 2B) which made up, on average, 47% and 5% of the sugars produced in the mature phase, respectively and 32% and 33% of the sugars produced in the senescent phase, respectively (Supplementary Fig. SS5). Mannuronic acid had the largest change in mole% of all sugars between the mature (5%) and senescent (33%) phases. In mature kelp blade mucilage, the molar percentage of glucosamine was significantly higher in the spring (13%) relative to the summer (3%), demonstrating the largest seasonal change (Fig. 2C; t-test, *P* < .001).

### Microbe-carbohydrate dynamics

**Figure 3:**
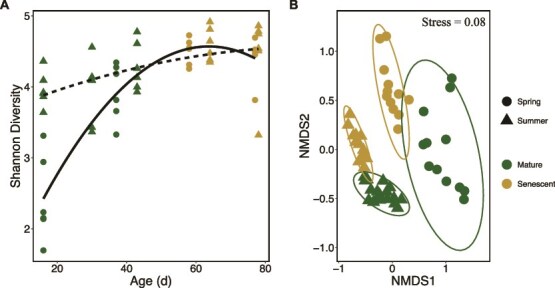
Giant kelp microbiome alpha and beta diversity between seasons and development stage. (A) Shannon diversity over age of the blade between spring (circles) and summer (triangles). Solid and dashed lines show the significant second order polynomial relationship between the giant kelp microbiome Shannon diversity and age in the spring and summer cohorts, respectively. (B) NMDS plot showing significant difference in the microbial community composition between seasons and development stage. NMDS ordinations used Bray–Curtis dissimilarities from arcsine-transformed 16S rRNA amplicon relative abundances. The legend applies to both panels.

**Figure 4:**
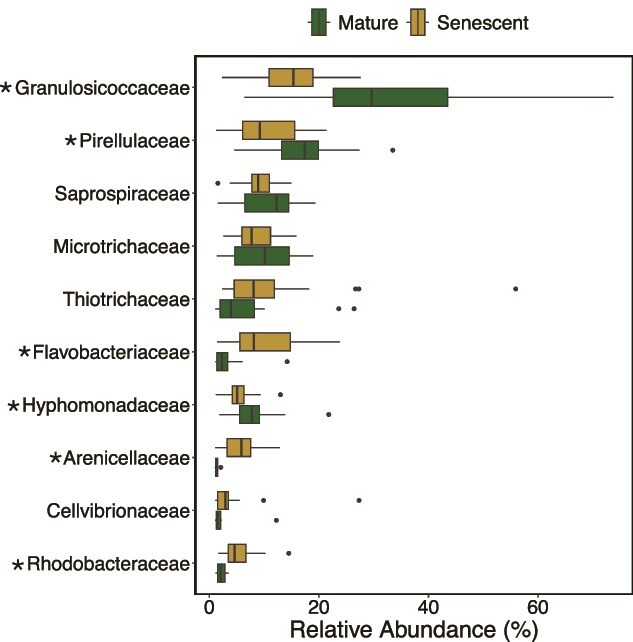
Relative abundance of bacterial clades at the family level on the surface of giant kelp blades between mature and senescent developmental stages. Shown are the top 10 families with a collective relative abundance >1% during either developmental stage. The left and right border of each box represent the 25th and 75th percentiles, the vertical line inside each box represents the median and the whiskers represent 1.5 × interquartile range (IQR). Outliers are shown by points beyond the whiskers. Asterisks next to family names indicate significant differences in the relative abundances between developmental stages (ANOVA, FDR-adjusted *P*-value < .05).

Coincident with altered mucilage carbohydrate composition, we observed significant differences in the giant kelp microbiome between seasons and developmental stages. In both seasons, we observed a significant increase in bacterial diversity with age (Ordinary Least Squares regression; Spring: *R*^2^ = 0.82, *P* < .001; Summer: *R*^2^ = 0.23, *P* = .01) although the spring cohort had lower initial diversity (Fig. 3A). Multivariate analysis demonstrated that the composition of the giant kelp microbiome was significantly different between season (PERMANOVA, *R*^2^ = 0.26, *P* < .001) and developmental stage (PERMANOVA, *R*^2^ = 0.18, *P* < .001) and there was a significant interaction between season and developmental stage (Fig. 3B; Two-way PERMANOVA, *R*^2^ = 0.10, *P* < .001). Family-level shifts between mature and senescent kelp were largely driven by a significant increases in *Flavobacteriacea*, and *Arenicellaceae*, *and Rhodobacteraceae* and a significant decreases in Granulosicoccaceae, *Pirellulaceae, and Hyphomonadaceae* (Fig. 4, analysis of variance (ANOVA), FDR-adjusted *P*-value <.05,Supplementary Table SS1). Family level changes were less pronounced between seasons (Supplementary Fig. SS6, Supplementary Table SS2). At the ASV level, we identified 371 unique taxa that varied significantly in relative abundance between season or developmental stage, most of which were from the phyla Actinobacteriota, Bacteroidota, Planctomycetota, Pseudomonadota (Gammaproteobacteria in particular), and Verrucomicrobiota (FDR-adjusted *P*-values < .01, DEseq2, Supplementary Data SS3 and SS4). Of these 371 ASVs, 44 had a relative abundance >1% in more than 10% of samples. We consider these 44 ASVs to be abundant and common microbial taxa in the giant kelp microbiome. Hierarchical clustering was used to identify relationships between these 44 abundant ASVs and the mole% of sugars produced by giant kelp based on Spearman’s rank correlation coefficients.

**Figure 5:**
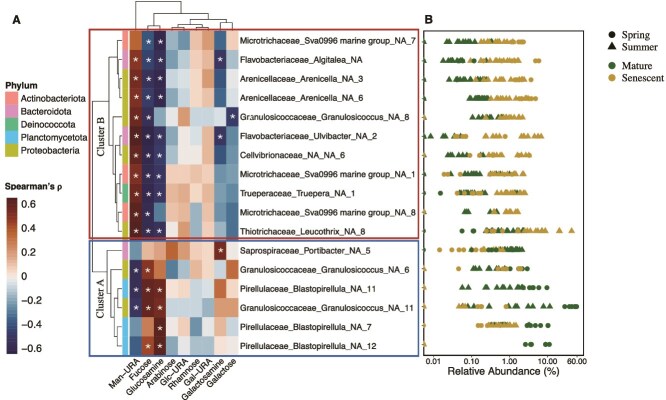
Abundant microbes are differentially correlated to the sugar content of giant kelp mucilage. (A) Heatmap showing the Spearman’s rank correlation coefficient between the relative abundance of differentially abundant ASVs (*n* = 17) with at least one significant positive or negative correlation (Spearman’s rank correlation, $\rho$ > 0.5, FDR-adjusted *P*-value < 0.05) with the mole% of a sugar monomer in giant kelp mucilage. The strength and direction of the Spearman’s ranked correlation are shown by the color map. Significant correlations are show by the asterisks. Hierarchical clustering shows two main clusters of ASV-sugar correlations. Cluster A is shown in the bottom blue rectangle, and Cluster B is shown in the top red rectangle. The phylum of each ASV is shown by the row color at the tip of each branch. Glc-URA, glucuronic acid; Gal-URA, galacturonic acid; Man-URA, mannuronic acid. (B) Range in the relative abundance (log scale) of each differentially abundant taxa in all kelp microbiome samples across season and physiological state.

For 17 of the 44 ASVs, there was a significant correlation with the mole% of at least one sugar in giant kelp mucilage (Spearman’s rank correlation, $\rho$ > 0.5, FDR-adjusted *P* < .05, Supplementary Table SS3 and SS4). These ASVs grouped into two clusters, primarily based on their correlation with fucose, glucosamine, and mannuronic acid (Fig. 5A). The first cluster (Cluster A) included ASVs whose relative abundance was positively correlated with mucilage rich in fucose and/or glucosamine or negatively correlated with mannuronic acid (Supplementary Table SS3). Members of the family *Pirellulaceae* from the phylum Planctomycetota accounted for three of the four ASVs in Cluster A. Cluster A also contained two ASVs from the family *Granulosicoccaceae* in the phylum Pseudomonadota (Fig. 5A).

The second cluster (Cluster B) was characterized by a significant positive relationship between relative abundances and mucilage rich in mannuronic acid and/or a negative correlation with mucilage rich in fucose and/or glucosamine (Supplementary Table SS4). This cluster included members of the families *Flavobacteriaceae, Cellvibrionaceae, Arenicellaceae, Trueperaceae, Thiotrichaceae, and Granulosicoccaceae* (Fig. 5A).

### Planctomycetota growth on model carbohydrates

**Figure 6:**
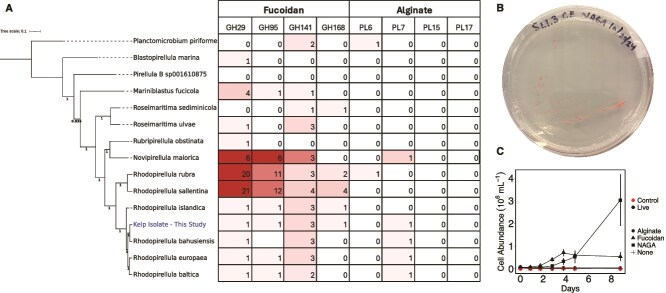
The Planctomycetota family *Pirellulaceae* can metabolize fucoidan, but not alginate. (A) Phylogenomic tree of Planctomycetota isolates within the *Pirellulaceae* family. The strain isolated in this study is highlighted in blue text as “Kelp Isolate – This Study”. The tree was rooted with one sequence from the Planctomycetota order Planctomycetales (family *Planctomycetaceae*), located at the top of the tree. Bootstrap scores are shown at the nodes. Paired with the phylogenomic position of each isolate are the number of GH (GH29, GH95, GH141, GH168) and polysaccharide lyases (PL6, PL7, PL15, PL17) involved in fucoidan and alginate degradation, respectively, found in their genomes. (B) Isolation of *Rhodopirellula* sp. on an agar plate. (C) Isolate growth on model carbohydrates. Control incubations are sterile incubation media with all carbohydrate amendments (shapes). NAGA is *N-*acetyl glucosamine. Live incubations show the isolate’s growth capability in response to amended carbohydrates. Points and error bars are the mean ± 1 SD cell abundance from triplicate incubations.

We cultivated a bacterial isolate from the surface of a giant kelp blade belonging to the phylum Planctomycetota (family *Pirellulaceae,* genus *Rhodopirellula*) (Fig. 6A and B) that we used in subsequent growth assays on model carbohydrates*.* We confirmed the ability of *this Rhodopirellula sp*. to grow on different carbohydrates, including those from kelp (Fig. 6C). We observed no growth in our sterile media controls, or the carbohydrate amended media without cells. In treatments with the isolate added, we observed no cell growth in unamended treatments. The isolate grew on both *N*-acetyl glucosamine and fucoidan, but not alginate.

In our phylogenomic analysis of the family *Pirellulaceae,* we found that carbohydrate-active enzymes associated with fucoidan metabolism, including the glycoside hydrolases (GH) GH29, CH95, GH141, and GH168 were common and abundant in the genomes of this family (Fig. 6A). Further, we found that only half of the isolates contained a copy of either the alginate lyases PL6 or PL7 and none contained copies of the oligoalginate lyases PL15 or PL17, the enzymes needed to metabolize oligoalgiantes into sugar monomers for metabolism (Fig. 6A). From the antiSMASH analysis, we found that our isolate may produce a number of secondary metabolites, such as terpenes, non-ribosomal peptides, and polyketides (Supplementary Fig. SS7). It was also predicted to synthesize the algicidal compound 3,5-dibromo-p-anisic acid [71].

## Discussion

### Nutrient availability and age shape kelp physiology

Giant kelp physiology between and within cohorts varied with seasonal nitrogen availability and age-related senescence. At our study site, giant kelp growth is primarily limited by inorganic nitrogen availability, which is depleted in the summer, but is replenished in the spring due to coastal upwelling (Fig. 1) [72, 73]. Giant kelp adjusts to periods of low nitrogen availability by adjusting its tissue nitrogen content, resulting in elevated tissue C:N ratios [72] . In our study, the average percent tissue-N of mature giant kelp in the summer (0.91% ± 0.06%) was below the minimum value (0.98%) recorded in the Santa Barbara Coastal Long Term Ecological Research (SBC LTER) time-series of kelp at our study site, whereas the percent tissue-N in the spring was, on average (2.6% ± 0.14%), equal to the average for our study site (2.7% ± 0.8%) [73]. In this context, our summer sampling captured kelp in a period of nitrogen depletion (tissue-N < 1%) which is suggested to result in diminished growth rates and kelp survival [33].

In addition to seasonal fluctuations in physiology, giant kelp undergoes progressive senescence, a rapid decline in physiological condition in response to age [74]. Our results align with previous studies indicating that senescence occurs independently of environmental conditions and begins when kelp tissue age exceeds ~50 days [30], potentially in response to declining photosynthetic performance [75, 76]. These age-related physiological changes were associated with a decrease in tissue-N (Fig. 2A) and changes to the composition of mucilage released by giant kelp (Fig. 2B and C).

### Giant kelp microbiome composition

The relative abundance of major epibiont bacterial families at different stages of kelp development is broadly consistent with observations of kelp microbiomes over tissue age and between healthy and stressed or senescent tissue (Fig. 4) [37, 42, 43, 77, 78]. Likewise, the increase in diversity of the microbiome with age is consistent with previous observations [77]; however, to our knowledge this is the first report of significantly lower Shannon diversity in young kelp in the spring compared to the summer (Fig. 3A). It has been proposed that the kelp microbiome is established through environmental acquisition over vertical transmission [79]. Therefore, the composition of kelp’s extracellular mucilage layer likely plays a major role in the establishment of the kelp microbiome. Kelps continuously shed and replace tissue year-round [80] and factors that affect mucilage composition, such as nitrogen availability, may have consequences for the biofilm composition of new tissue, as has been proposed for developing terrestrial plant rhizospheres [8].

### Exudate and microbiome composition dynamics

Simultaneous changes in the carbohydrate composition of kelp mucilage and microbial taxa suggest an important role for mucilage composition in shaping the kelp microbiome. These associations further revealed that extrinsic factors, such as nitrogen availability, and intrinsic factors such as development stage regulate the composition of the giant kelp mucilage. Notably, we observed an enrichment of glucosamine, a nitrogen-containing sugar, in mucilage produced by mature kelp in the spring compared to the nitrogen-depleted kelp in the summer (Fig. 1C). A similar pattern has been observed in the root exudates of plants, whereby the addition of inorganic nitrogen results in the enhanced release of nitrogen-containing metabolites relative to roots grown under nitrogen-limiting conditions [8]. Baker *et al.* [8] found that the release of nitrogen-rich metabolites by plant roots selected for a less diverse and unique microbial community compared to plants grown in nitrogen-limited conditions, a pattern we also observed in the kelp microbiome (Fig. 2). Glucosamine is a common sugar in the mucus produced by many organisms, including corals and humans [16, 19, 81], and has been shown to serve an important role in regulating attachment and biofilm formation by bacteria [82, 83]. Because kelp mucilage is constantly sloughed into the surrounding seawater, it must be replaced to maintain its function in the extracellular matrix. Therefore, the cost of exuding nitrogen-containing compounds that must be continuously replaced may be too high to be maintained under nitrogen-depleted conditions experienced by giant kelp in the summer.

Fucose was the most abundant sugar in the carbohydrates released by mature giant kelp in both seasons, consistent with previous studies of brown macroalgae [18, 51, 52]. The presence of fucose in brown macroalgal mucilage is due to the secretion of fucoidan, a water-soluble, sulfated polysaccharide that is deposited onto the exterior of the thallus through secretory canals to build the kelp’s extracellular mucilage, which is continuously sloughed into the surrounding seawater [84, 85]. Fucoidan lacks nitrogen which may explain its consistent dominance in kelp mucilage across a range of tissue-N content. Several studies document the potential defensive properties of fucoidans [69, 82, 86] and its proposed defensive role is supported by its observed recalcitrance to bacterial degradation [87].

In both seasons, there was an increase in the mole% of mannuronic acid in mucilage as kelp blades aged and senesced (Fig. 2B, Supplementary Fig. SS5). Mannuronic acid is an indicator of the macroalgal polysaccharide alginate, which comprises up to half of kelp biomass [88]. Microbial taxa positively correlated to the mole% of mannuronic acid included members of the family *Flavobacteriaceae* and several Gammaproteobacteria, including one member of the family *Thiotrichaceae* (Fig. 5A, Cluster B). Flavobacteriia and Gammaproteobacteria are archetypal fast-growing carbohydrate degraders with adaptations that enable them to grow attached to surfaces and are enriched in alginate and oligoalginate lyases [89–92]. Using DNA stable isotope probing with ^13^C-labeled alginate, Thomas *et al.* [89] demonstrated that the main consumers of alginate in coastal seawater were bacteria from the families *Flavobacteriaceae*, *Alteromonadaceae*, and *Thiotrichaceae* clades. The relative abundance of *Flavobacteriaceae* and *Thiotrichaceae* in kelp biofilms is typically higher on stressed or senescent kelp tissue [37, 78] as observed in this study (Fig. 4), and in the early stages of kelp biomass decay these groups dominate alginate hydrolysis [90].

From our observations, we hypothesize that as kelp begins to senesce, the release of alginate is initiated by alginate degrading “pioneer” microbes whose alginate lyases release alginate oligomers, facilitating the growth of alginate “harvesters” and “scavengers” [93]. This dynamic would explain the enhanced solubilization of kelp tissue with age that we observed in a complementary study [75]. Notably, these potential alginate degraders (i.e. *Flavobacteriaceae* and *Thiotrichaceae*) were observed at low relevant abundance in the mature kelp microbiome (Fig. 5B), suggesting some process maintained their growth until kelp entered its senescent phase. The mechanisms of kelp senescence are not well understood; however, senescence of giant kelp blades in both seasons occurred after 50 days of age, following a linear decline in photosynthetic rates (see our complementary study [75]), suggesting intrinsic regulation of senescence [94]. Although we observed signs of intrinsic regulation of senescence, we cannot completely rule out that associated bacteria trigger senescence. For example, AntiSMASH analysis of our Planctomycetota isolate showed the potential for the production of 3,5-dibromo-p-anisic acid, a potent algal toxin produced by other strains of Planctomycetota [71] (Supplementary Fig. SS7). Planctomycetota may have a “Jeckyll and Hyde” behavior with macroalgae and trigger or enhance senescence in response to declining algal physiological condition with age [95].

### Potential mechanisms behind carbohydrate-microbe associations

Without further study of their native composition, we can only speculate about the structure and function of the carbohydrates in giant kelp mucilage. However, the role of carbohydrates secreted to the outer membrane layers of animals and phytoplankton is well established. For example, in bacterial biofilm formation, carbohydrates tethered to the cell surface of a host serve as recognition sites for carbohydrate-protein binding proteins (lectins) that act in concert with larger attachment proteins (adhesins). These lectin-adhesin complexes mediate the attachment of bacteria, including pathogens to the host surface [96]. In a model bacterium-diatom association, the lectin MpPA14 exhibits a strong binding affinity for fucose and *N*-acetyl glucosamine residues [82]. Guo *et al.* [82] demonstrated that MpPA14 and the MpPA14 lectin homolog from *Vibrio cholera* were inhibited and unable to bind to diatom cells when exposed to high concentrations of free fucose. A strong binding affinity of MpPA14 was also observed for fucoidan, a fucose-rich polysaccharide released by brown macroalgae. These data indicate a potentially conserved defensive role of fucose-containing carbohydrates exuded by members of the Stramenopile lineage, such as diatoms and kelps.

Giant kelp may release high concentrations of fucose-rich carbohydrates to disrupt biofilm formation by potential pathogens. Indeed, all taxa from Cluster B (Fig. 4A), which includes several taxa associated with macroalgal tissue degradation, such as Flavobacteriia, exhibited a negative correlation with the fucose content of the exuded carbohydrates (Spearman’s ranked correlations, FDR-adjusted *P* < .05; Supplementary Table SS4). This was surprising, as previous metagenomic-based studies predict that members of the Bacteroidota phylum, especially members of *Flavobacteraceae*, are specialized in degrading fucoidans [97, 98], however this family was observed at relatively low relative abundances in mature kelp biofilms (Figs. 4 and 5), when fucose dominated the mucilage sugar content (Fig. 2B). Therefore, other factors, in addition to carbohydrate substrate preferences may contribute to the structure of the kelp microbiome; possible factors could be the production of secondary metabolites by Planctomycetota [63, 99] or the anti-adhesion property of fucoidan [82]. Regarding the former, AntiSMASH analysis of our isolated *Rhodopirellula* genome annotated two terpene biosynthetic gene clusters, two type I polyketide synthases (PKS), one type III PKS, and hybrid type I PKS/terpene and type I/non ribosomal peptide synthases (Supplementary Fig. SS7). This is consistent with other isolates from the *Pirellulaceae* family [63] and suggests their abundances in kelp biofilms may be due to a combination of their ability to degrade fucoidan (Fig. 6C) and their production of defensive secondary metabolites. Experiments to address secondary metabolite production by this isolate are currently underway.

Glucosamine may serve as an important carbon and nitrogen source for the growth of microbes beneficial to giant kelp. In the marine environment, glucosamine is more commonly found in its acetylated form (*N*-acetyl glucosamine) [100]. Our carbohydrate measurements require an acid hydrolysis step, which cleaves the acetyl group resulting in glucosamine. Therefore, our glucosamine measurements reflect the contribution of both *N*-acetyl glucosamine and glucosamine residues. The microbial taxa that positively correlated with glucosamine included members from the family *Pirellulaceae* within Planctomycetota, and the *Granulosicoccaceae* family of Pseudomonadota (Cluster A, Fig. 5A, Supplementary Table SS3). Cultured strains of Planctomycetota such as *Pirellula* sp. strain 1 can metabolize *N*-acetyl glucosamine as their sole carbon and nitrogen source [101], and in a comparison of 79 cultured members of Planctomycetota, *N*-acetyl glucosamine was found to be the most efficient carbon source for their isolation [63]. Moreover, previous work suggests *N*-acetyl glucosamine may trigger biofilm formation by some members of Planctomycetota*,* such as *Rhodopirellua baltica* [102].

Planctomycetes are dominant members of healthy macroalgal microbiomes [37, 43, 103] and it has been proposed they alter the composition of biofilms through the production of secondary metabolites such as stieleriacines [99]. We demonstrate that a *Rhodopirellula* strain, isolated from the surface of a giant kelp blade, can grow on fucoidan and *N-*acetyl glucosamine, but not alginate (Fig. 6C). This carbohydrate substrate preference is consistent with our observed correlations between the relative abundance of members of the Planctomycetota family *Pirellulaceae* in the kelp microbiome and the sugar content of giant kelp mucilage (Fig. 5A, Cluster A). Although only one isolate was tested in this study, the ability for growth on fucoidan but not alginate is broadly consistent with the metabolic potential of other isolated members of the family *Pirellulaceae* (Fig. 6A). Further, several recent studies also confirm that the isolates from the *Pirellulaceae* family degrade fucoidan [40, 104]. We also note that even though some *Pirellulaceae* genomes, including our isolate genome, contained copies of the alginate lyases PL6 or PL7, none contain the oligoalginate lyases PL15 or PL17, which degrade oligoalginates into monomers for metabolism (Fig. 6A) [93]. We hypothesize that exudation of specific carbohydrates, such as fucoidan serves to enrich their microbiome in commensal or beneficial microbes such as Planctomycetota that do not degrade important structural polymers like alginate. Such carbohydrate-microbe synergy is consistent with observations of plant rhizospheres, corals, and the human gut [14, 16, 81, 105–107].

## Supplementary Material

Supplementary_Material_ycaf197

Supplemental_Data_1_ycaf197

Supplemental_Data_2_ycaf197

Supplemental_Data_3_ycaf197

Supplemental_Data_4_ycaf197

## Data Availability

DNA sequence data are available in the National Center for Biotechnology Information Sequence Read Archive under PRJNA1256130. Whole genome sequence data for the *Rhodopirellula sp*. isolate used in this study can be found under PRJNA1314851. R scripts for analysis can be found at https://github.com/chance-english.
