# Supplementary material for: Seasonal and developmental stage changes in mucilage carbohydrate content shape the kelp microbiome
Source: ISME Commun. 2025 Oct 29;5(1):ycaf197. doi: 10.1093/ismeco/ycaf197 (PMC12642873; doi:10.1093/ismeco/ycaf197)
Supplement: Supplementary_Material_ycaf197 [file supplementary_material_ycaf197.docx]

**Supplemental Material**

**Seasonal and developmental stage changes in mucilage carbohydrate content shape the kelp microbiome**

*Chance J. English*^*1,2^*, Meenakshi Manoj^1^, Lillian C. Henderson^1^, Keri Opalk^1^, Craig A. Carlson*^1,3^

1. Marine Science Institute, University of California Santa Barbara, CA, USA
2. Earth Research Institute, University of California, Santa Barbara, CA, USA
3. Department of Ecology, Evolution and Marine Biology, University of California, Santa Barbara, CA, USA

* Corresponding author: Chance J. English

Email: [cje@ucsb.edu](mailto:cje@ucsb.edu)

(951) 286-8416

Supplemental Table 1: ANOVA results for differences in relative abundance of top 10 families btween mature and developmental stages shown in Figure 4. Significant results means the observed relative abundances of the family was higher in in the mature developmental stage

| **family** | **term** | **df** | **sumsq** | **meansq** | **statistic** | **p.value** | **adjusted_p.value** |
| --- | --- | --- | --- | --- | --- | --- | --- |
| Granulosicoccaceae | phys_state | 1 | 0.38 | 0.38 | 20.47 | 3.65E-05 | 3.65E-04 |
| Thiotrichaceae | phys_state | 1 | 0.03 | 0.03 | 2.82 | 0.100 | 0.144 |
| Pirellulaceae | phys_state | 1 | 0.07 | 0.07 | 17.75 | 1.01E-04 | 5.03E-04 |
| Cellvibrionaceae | phys_state | 1 | 0.00 | 0.00 | 0.23 | 0.638 | 0.638 |
| Flavobacteriaceae | phys_state | 1 | 0.04 | 0.04 | 17.37 | 1.71E-04 | 5.71E-04 |
| Hyphomonadaceae | phys_state | 1 | 0.01 | 0.01 | 7.99 | 0.007 | 0.011 |
| Saprospiraceae | phys_state | 1 | 0.00 | 0.00 | 1.21 | 0.277 | 0.344 |
| Microtrichaceae | phys_state | 1 | 0.00 | 0.00 | 1.06 | 0.309 | 0.344 |
| Rhodobacteraceae | phys_state | 1 | 0.01 | 0.01 | 16.11 | 2.71E-04 | 6.78E-04 |
| Arenicellaceae | phys_state | 1 | 0.01 | 0.01 | 15.72 | 4.03E-04 | 8.06E-04 |

Supplemental Table 2: ANOVA results for differences in relative abundance of top 10 families between spring and summer cohorts shown in Supplemental Figure 6. Significant results means the observed relative abundances of the family was higher in the spring.

| **family** | **term** | **df** | **sumsq** | **meansq** | **statistic** | **p.value** | **adjusted_p.value** |
| --- | --- | --- | --- | --- | --- | --- | --- |
| Granulosicoccaceae | season | 1 | 0.045 | 0.045 | 1.79 | 0.1870 | 0.255150 |
| Thiotrichaceae | season | 1 | 0.009 | 0.009 | 0.90 | 0.3487 | 0.387435 |
| Pirellulaceae | season | 1 | 0.009 | 0.009 | 1.92 | 0.1721 | 0.255150 |
| Cellvibrionaceae | season | 1 | 0.006 | 0.006 | 1.73 | 0.2041 | 0.255150 |
| Flavobacteriaceae | season | 1 | 0.036 | 0.036 | 14.10 | 0.0006 | 0.001930 |
| Hyphomonadaceae | season | 1 | 0.000 | 0.000 | 0.08 | 0.7820 | 0.781972 |
| Saprospiraceae | season | 1 | 0.056 | 0.056 | 52.15 | 0.0000 | 0.000000 |
| Microtrichaceae | season | 1 | 0.037 | 0.037 | 20.60 | 0.0000 | 0.000203 |
| Rhodobacteraceae | season | 1 | 0.007 | 0.007 | 10.69 | 0.0023 | 0.005728 |
| Arenicellaceae | season | 1 | 0.002 | 0.002 | 1.71 | 0.2005 | 0.255150 |

**Supplemental Table 3.** Spearman’s rank correlation rho and FDR-adjusted p-values for abundant ASVs and sugar mole% in Cluster A (Figure 5A).

| **Sugar** | **Pirellulaceae_Blastopirellula_NA_12** | **Pirellulaceae_Blastopirellula_NA_7** | **Granulosicoccaceae_Granulosicoccus_NA_11** | **Pirellulaceae_Blastopirellula_NA_11** | **Granulosicoccaceae_Granulosicoccus_NA_6** | **Saprospiraceae_Portibacter_NA_5** |
| --- | --- | --- | --- | --- | --- | --- |
| Fucose | 0.42,0.04001 | 0.28,0.23828 | 0.58,0.00137 | 0.58,0.00131 | 0.48,0.01154 | 0.11,0.72229 |
| Rhamnose | -0.13,0.65786 | -0.24,0.31968 | -0.08,0.7988 | -0.1,0.74709 | 0.11,0.70898 | 0.06,0.85406 |
| Galactosamine | 0.02,0.95119 | 0.11,0.72086 | 0.19,0.45915 | 0.33,0.13624 | -0.08,0.7946 | 0.52,0.00553 |
| Arabinose | -0.2,0.44136 | 0,0.9889 | -0.08,0.79993 | 0.01,0.97272 | -0.29,0.19491 | 0.34,0.12517 |
| Glucosamine | 0.61,0.00082 | 0.62,0.00072 | 0.54,0.00364 | 0.55,0.00294 | 0.31,0.16831 | 0.18,0.50321 |
| Galactose | -0.01,0.97369 | -0.09,0.77111 | 0.2,0.41287 | 0.03,0.92888 | 0.32,0.14726 | 0,0.99919 |
| Gal-URA | -0.14,0.62703 | -0.03,0.93147 | -0.17,0.53314 | -0.06,0.84141 | 0.03,0.92321 | 0.04,0.91045 |
| Glc-URA | -0.05,0.8645 | 0.06,0.83048 | -0.1,0.73885 | 0.04,0.90437 | -0.13,0.6549 | 0.18,0.47232 |
| Man-URA | -0.38,0.08039 | -0.25,0.3076 | -0.51,0.00716 | -0.56,0.00232 | -0.56,0.00236 | -0.24,0.31968 |

**Supplemental Table 4.** Spearman’s rank correlation rho and FDR-adjusted p-value for abundant ASVs and sugar mole% in Cluster B (Figure 5A).

| **Sugar** | **Thiotrichaceae_Leucothrix_NA_8** | **Microtrichaceae_Sva0996 marine group_NA_8** | **Trueperaceae_Truepera_NA_1** | **Microtrichaceae_Sva0996 marine group_NA_1** | **Cellvibrionaceae_NA_NA_6** | **Flavobacteriaceae_Ulvibacter_NA_2** | **Granulosicoccaceae_Granulosicoccus_NA_8** | **Arenicellaceae_Arenicella_NA_6** | **Arenicellaceae_Arenicella_NA_3** | **Flavobacteriaceae_Algitalea_NA** | **Microtrichaceae_Sva0996 marine group_NA_7** |
| --- | --- | --- | --- | --- | --- | --- | --- | --- | --- | --- | --- |
| Fucose | -0.6, 0.00103 | -0.59, 0.00127 | -0.62, 0.00072 | -0.57, 0.00183 | -0.62, 0.00072 | -0.65, 0.00062 | -0.56, 0.00204 | -0.48, 0.01154 | -0.52, 0.00592 | -0.45, 0.02168 | -0.4, 0.05822 |
| Rhamnose | -0.16, 0.56818 | -0.18, 0.48463 | 0.04, 0.90403 | 0.08, 0.7963 | -0.01, 0.97369 | -0.06, 0.83943 | -0.09, 0.77111 | 0.04, 0.90403 | 0.04, 0.88867 | 0.07, 0.82261 | 0.1, 0.74264 |
| Galactosamine | -0.16, 0.56818 | -0.24, 0.33629 | -0.22, 0.39835 | -0.24, 0.32614 | -0.39, 0.06541 | -0.5, 0.00812 | -0.07, 0.80713 | -0.36, 0.10322 | -0.39, 0.06515 | -0.49, 0.0094 | -0.35, 0.11039 |
| Arabinose | -0.01, 0.98342 | 0.1, 0.74264 | 0.11, 0.72229 | 0.06, 0.8338 | -0.15, 0.60259 | -0.2, 0.44136 | -0.03, 0.93147 | -0.21, 0.41287 | -0.1, 0.73885 | -0.23, 0.36489 | -0.17, 0.50885 |
| Glucosamine | -0.53, 0.00428 | -0.31, 0.17494 | -0.51, 0.00687 | -0.52, 0.00617 | -0.49, 0.01013 | -0.51, 0.00716 | -0.21, 0.41287 | -0.61, 0.00074 | -0.6, 0.00094 | -0.55, 0.00294 | -0.62, 0.00072 |
| Galactose | -0.32, 0.15322 | -0.33, 0.13215 | -0.28, 0.23744 | -0.22, 0.37359 | -0.25, 0.31968 | -0.26, 0.28245 | -0.47, 0.01436 | -0.05, 0.86412 | -0.03, 0.94058 | -0.13, 0.64198 | 0.08, 0.79993 |
| Gal-URA | 0.07, 0.82261 | 0.08, 0.7988 | 0.11, 0.69992 | 0.08, 0.80234 | 0.13, 0.65786 | 0.12, 0.69992 | -0.09, 0.76834 | 0.1, 0.74085 | 0.19, 0.45589 | 0.12, 0.69874 | 0.19, 0.44427 |
| Glc-URA | 0.21, 0.41287 | 0.13, 0.65786 | 0.16, 0.56818 | 0.07, 0.82261 | -0.01, 0.97369 | -0.01, 0.98111 | 0.18, 0.47232 | -0.08, 0.79672 | -0.07, 0.82575 | -0.12, 0.6634 | -0.24, 0.33247 |
| Man-URA | 0.65, 0.00062 | 0.62, 0.00072 | 0.55, 0.00294 | 0.5, 0.00805 | 0.59, 0.00127 | 0.64, 0.00062 | 0.57, 0.00183 | 0.51, 0.00716 | 0.54, 0.00383 | 0.48, 0.01154 | 0.35, 0.11039 |


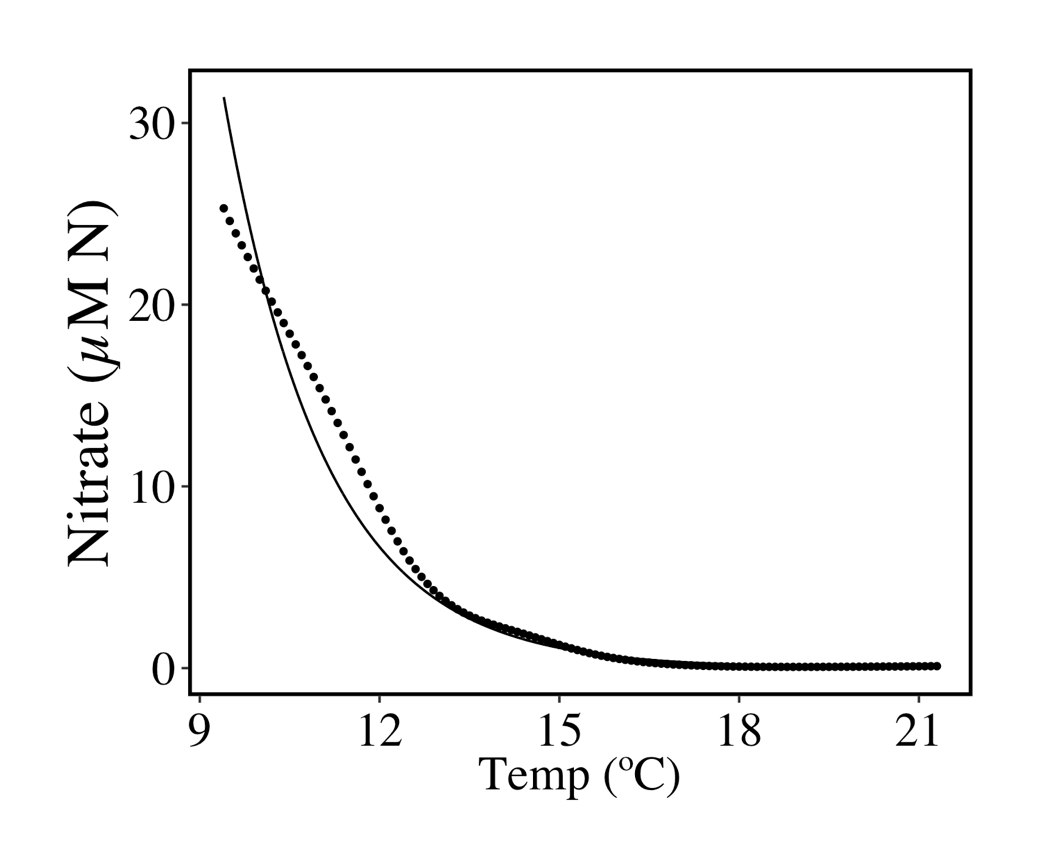


**Supplemental Figure 1**. Temperature to nitrate relationship for inshore water in the Santa Barbara Channel. Points are from the T2N look up table compiled by Snyder et al. 2020 . Solid line is the exponential regression used to calculate nitrate at Mohawk Reef at our study site: Nitrate (µM N) = 8565.5 * e^(-0.597*Temp(ºC))^, R^2^ = 0.95.

**Supplemental Figure 2**. ^1^H-^13^C HSQC NMR spectrum of IEX purified fucoidan from *Macrocystis pyrifera*. Correlations showing carbons with an even number of protons are blue and those with an odd number of protons are shown in red.

**
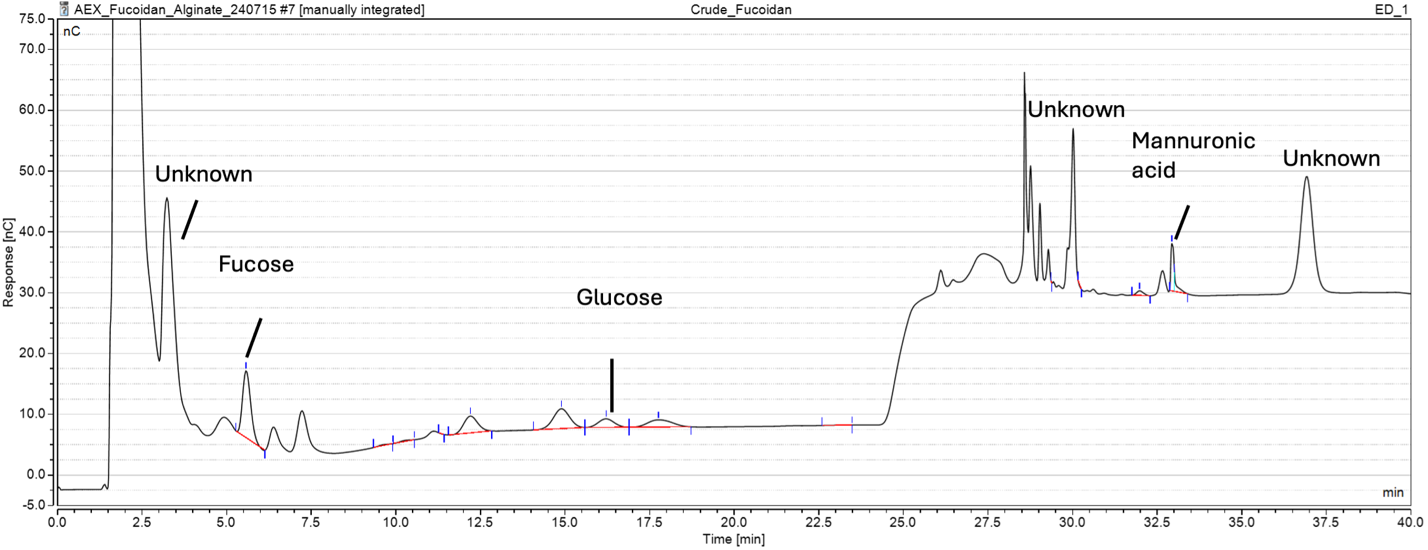
**

**Supplemental Figure 3.** HPAEC-PAD chromatogram of crude fucoidan from supplier. Note that peak area for fucose is relatively equal to areas from sugars not found in fucoidan (glucose, mannuronic acid) compared to fucose in IEX-purified fucoidan (Supplemental Figure 4). Also, there are large contributions from unknown peaks, possibly contaminating sugars in the sugar alcohol range (0 - 5 minutes) and acidic sugar range (30 - 40 minutes).

**
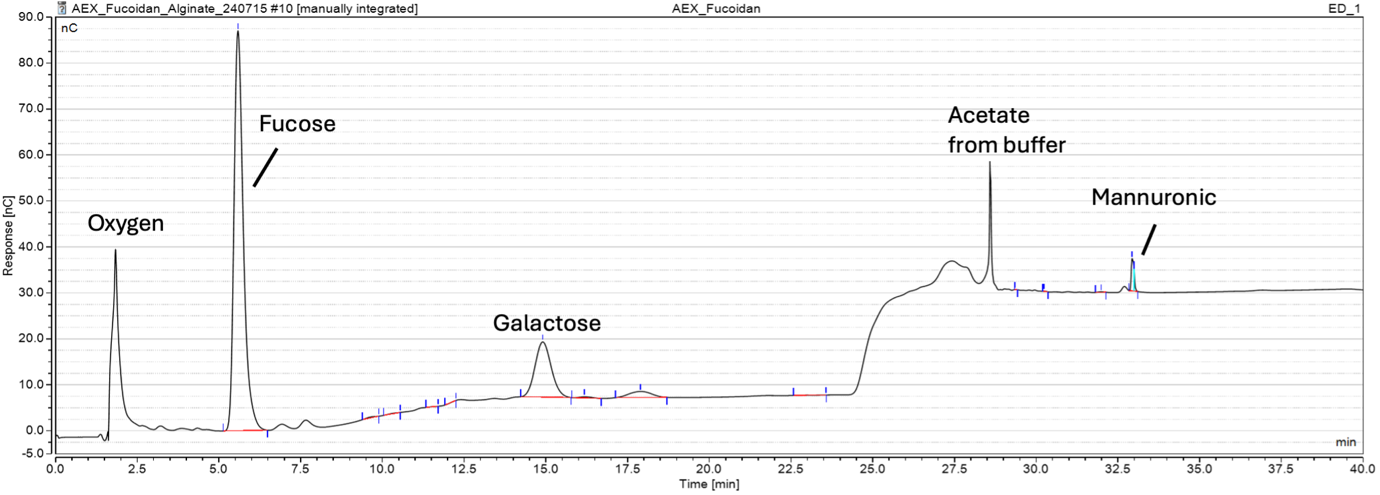
**

**Supplemental Figure 4.** HPAEC-PAD chromatogram of IEX purified fucoidan. Note that peak area of fucose enhanced compared to small remaining contaminating sugars (mannuronic acid) compared to Supplemental Figure 3. Contribution of galactose indicates fucoidan from *M. pyrifera* is likely a heterofucan. The two prominent peaks at the beginning and middle of the chromatogram are from dissolved oxygen and acetate from the sample and elution buffer, respectively.

**Supplemental Figure 5: Mole% of fucose and mannuronic acid (Man-URA) across age and and seasons. (A) Summer (B) Spring.** The top and bottom border of each box represent the 25^th^ and 75^th^ percentiles, the horizontal line inside each box represents the median and the whiskers represent 1.5*IQR. Outliers are shown by points beyond the whiskers.

Supplemental Figure 6: **Relative abundance of bacterial clades at the family level on the surface of giant kelp blades between the spring and summer periods**. Shown are the top 10 families with a collective relative abundance > 1% during either season. The left and right border of each box represent the 25^th^ and 75^th^ percentiles, the vertical line inside each box represents the median and the whiskers represent 1.5*IQR. Outliers are shown by points beyond the whiskers. Asterisks next to family names indicate significant differences in the relative abundances between seasons (ANOVA, FDR-adjusted p-value < 0.05).


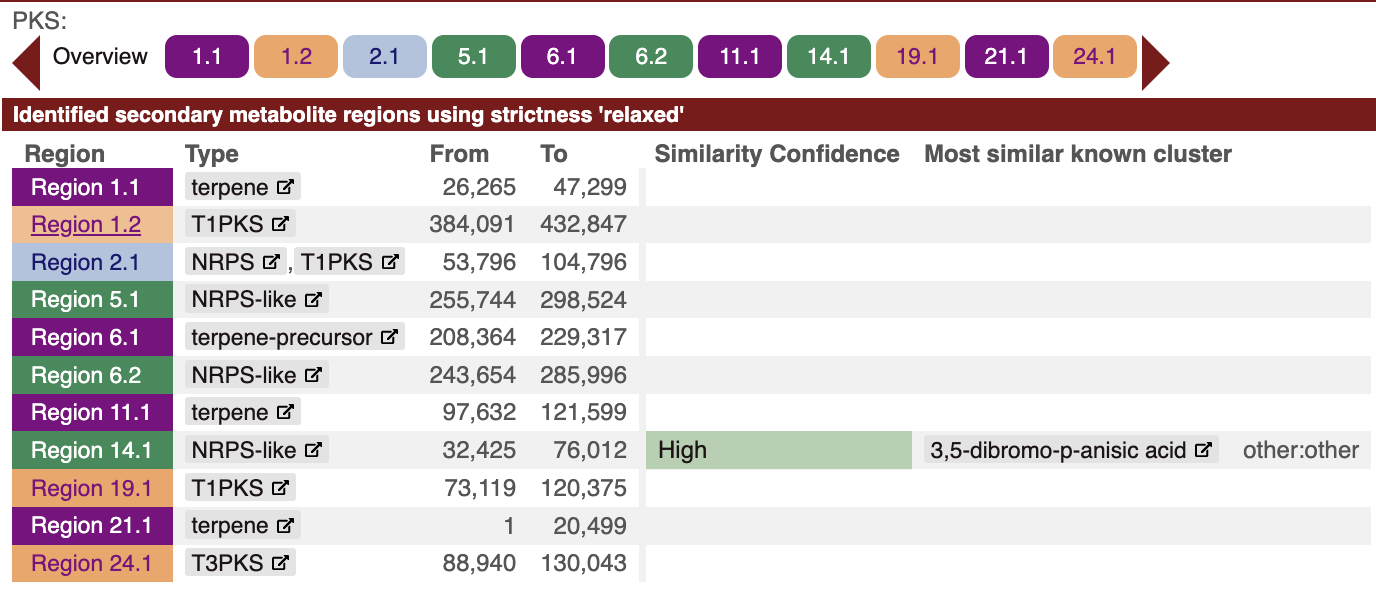


**Supplemental Figure 7: AntiSMASH analysis results of the *Rhodopirellula sp* isolate genome.**
